# Measurements, Thermodynamic Modeling, and a Hydrogen Bonding Study on the Solubilities of Metoprolol Succinate in Organic Solvents

**DOI:** 10.3390/molecules23102469

**Published:** 2018-09-26

**Authors:** Jian Shen, Xianrui Liang, Hao Lei

**Affiliations:** 1 School of Materials and Chemical Engineering, Ningbo University of Technology, Ningbo 315016, China; leihao94@sina.com; 2 College of Pharmaceutical Sciences, Zhejiang University of Technology, Hangzhou 310014, China; liangxrvicky@zjut.edu.cn

**Keywords:** metoprolol succinate, solubility, dissolution enthalpy, hydrogen bonding, thermodynamic model

## Abstract

The solubilities of metoprolol succinate (a cardioselective β1 adrenergic receptor) in methanol, ethanol, *n*-propanol, isopropanol, *n*-butanol, ethyl acetate, and acetone were measured at temperatures ranging from (278.2 to 318.2) K using a solid–liquid equilibrium method. The solubility of metoprolol succinate increases with increasing temperature. At a fixed temperature, the solubility decreases in the order methanol > ethanol > *n*-butanol > *n*-propanol > isopropanol > acetone > ethyl acetate. The enthalpy of fusion and the melting point of metoprolol succinate were determined by differential scanning calorimetry. The thermodynamic properties of the dissolution process, determined by a van’t Hoff analysis, have been obtained and are discussed. The modified Apelblat equation, Wilson model, and non-random two-liquid (NRTL) model were employed to correlate the solubilities of metoprolol succinate in different solvents. Finally, a quantitative structure–property relationship (QSPR) study of physical properties of solvents and density functional theory simulations of hydrogen-bonding structure were carried out to give the explanation for the sequence of solubility in alcohols. The density functional theory (DFT) calculations well illustrated that the solubility of metoprolol succinate in various alcohols can be mainly attributed to the intra- and intermolecular hydrogen bonds in metoprolol succinate-solvent complexes.

## 1. Introduction

Metoprolol succinate (C_34_H_56_N_2_O_10_, CAS no. 98418-47-4) is a selective β1-receptor blocker medication used to treat chest pain (angina), heart failure, and high blood pressure [1]. It is also used to prevent further heart problems after myocardial infarction, and to prevent headaches in those with migraines [2]. Impurities are easily introduced during the synthesis of metoprolol succinate [3] or during storage [4], which significantly affect its quality, safety, and efficacy.

The crystallization step plays an important role in the purification of intermediates and the final pharmaceutical ingredients [5,6]. The solubility of the target compound in different solvents [7,8,9] is required for the crystallization process. In addition, solubility data for metoprolol succinate in different solvents is also beneficial for analytical [10], inclusion [11], molecular thermodynamic, and interaction studies [12]. In recent years, physical–chemical properties such as the density, viscosity, and refractive index of aqueous solutions of metoprolol succinate have been studied [13]. However, the solubilities of metoprolol succinate in different solvents, including the most commonly used alcohol solvents, have not been reported.

In this work, the solubilities of metoprolol succinate in methanol, ethanol, *n*-propanol, isopropanol, *n*-butanol, ethyl acetate, and acetone at temperatures ranging from (288.2 to 318.2) K at atmospheric pressure were determined. The enthalpy of fusion and the melting temperature of metoprolol succinate were measured by differential scanning calorimetry (DSC). The thermodynamic properties of the dissolution process including the apparent Gibbs free energy, enthalpy, and entropy of solution were calculated by using the van’t Hoff equations. The modified Apelblat equation, Wilson model, and non-random two-liquid model (NRTL) model [14,15] were used to correlate the experimental solubilities of metoprolol succinate in different solvents. A simple quantitative structure–property relationship (QSPR) model between the physical properties of the alcohol solvents, and the solubilities of metoprolol succinate in these solvents has been tested. Finally, density functional theory (DFT) simulations of hydrogen-bonding structure were investigated to interpret the sequence of solubility in the selected alcohol solvents.

## 2. Results

### 2.1. Melting Point and Enthalpy of Fusion

**Figure 1:**
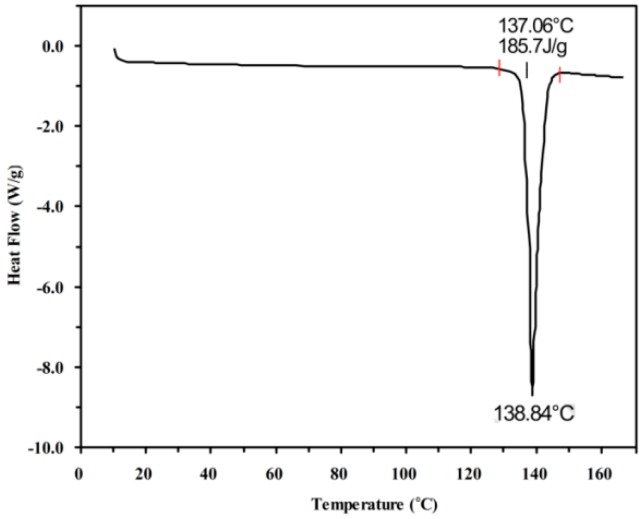
The differential scanning calorimetry (DSC) thermogram of metoprolol succinate.

The DSC thermogram is shown in Figure 1. The melting temperature (*T*_m_) and enthalpy of fusion (Δ*H*_fus_) of metoprolol succinate were found to be 137.0 ± 0.4 °C and 121.3 ± 0.1 kJ mol^−1^, respectively. The results are close to the reported *T*_m_ and Δ*H*_fus_ values of 137.8 °C and 118.2 kJ mol^−1^, respectively [16]. The entropy of fusion (Δ*S*_fus_) of metoprolol succinate was calculated using a thermodynamic relationship, and it was found to be 295.5 J K^−1^ mol^−1^.

### 2.2. Solubilities

The mole fraction solubilities of metoprolol succinate in different solvents at temperatures of (288.2, 293.2, 298.2, 303.2, 308.2, 313.2, and 318.2) K are listed in Table 1. The mole fraction solubilities are the average values of at least two measurements. The uncertainties (±) for each experimental value are also given in the table.

### 2.3. Thermodynamic Functions of Dissolution

The apparent enthalpy of dissolution (Δ*H*_sol,apparent_) was determined by van’t Hoff analysis, and it can be calculated using Equations (1)–(3):

(1) $$\left(\frac{\partial \ln x_{1}}{\partial \left(1/T-1/T_{hm}\right)}\right)\;=\;-\frac{\Delta H_{\mathrm{sol},\mathrm{apparent}}}{R}$$

(2) $$\Delta G_{\mathrm{sol},\mathrm{apparent}}\;=\;-R\;\times \;T_{\mathrm{hm}}\;\times \;\mathrm{intercept}$$

(3) $$\Delta S_{\mathrm{sol},\mathrm{apparent}}\;=\;\frac{\left(\Delta H_{\mathrm{sol},\mathrm{apparent}}\;-\;\Delta G_{\mathrm{sol},\mathrm{apparent}}\right)}{T_{\mathrm{hm}}}$$

**Figure 2:**
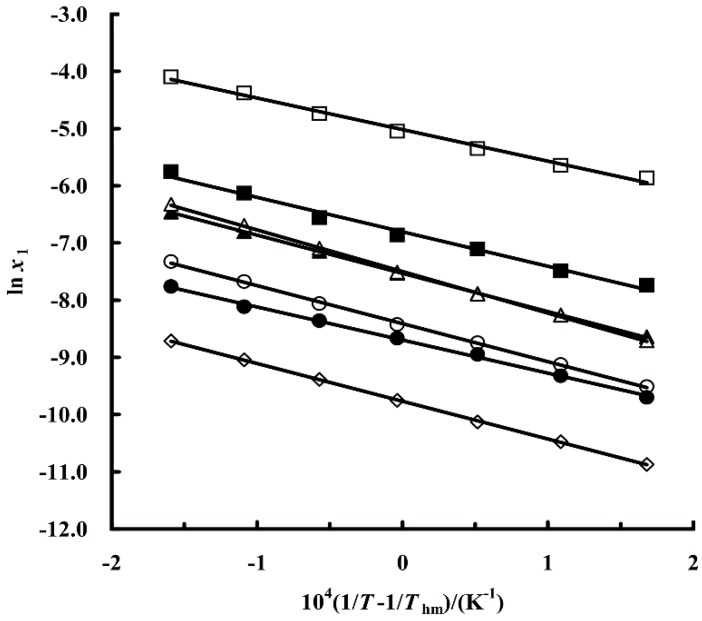
Modified van’t Hoff plot of the mole fraction solubility of metoprolol succinate in selected solvents: (□) methanol; (■) ethanol; (∆) *n*-propanol; (▲) isopropanol; (○) *n*-butanol; (●) ethyl acetate; (◊) acetone.

Here, *T*_hm_ is the mean harmonic temperature, and the value of *T*_hm_ obtained in this study is 302.87 K. Thus, treating the values of ln(*x*) as a linear function of 1/*T* − 1/*T*_hm_, as shown in Figure 2, the values of Δ*G*_sol,apparent_ and Δ*H*_sol,apparent_ can be obtained from the intercept and slope of the line, respectively. The apparent enthalpy, entropy, and the Gibbs free energy of the dissolution process are shown in Table 2.

### 2.4. Phase Equilibrium Models and Correlations

The temperature dependence of the drug solubility in a solvent can be modeled by empirical correlation [17,18], and by the activity coefficient method [19,20]. To select an appropriate model to describe the solubility of metoprolol succinate in the tested solvents, the experimental solubility data were correlated using three models: the modified Apelblat equation [21], the Wilson model [22], and the NRTL model [23].

#### 2.4.1. Modified Apelblat Equation

The modified Apelblat equation that is derived from the Clausius-Clapeyron model is shown in Equation (4) [18]:

(4) ln(x) = A + B/T + C lnT

Here, *T* is the solution temperature, *A*, *B*, and *C* are the equation parameters, and *x* is the mole fraction of the drug in the solution.

#### 2.4.2. Wilson Model

The solubility dependence on the temperature in various solvents can be described as:

(5) $$\ln\left(x_{i}\cdot \gamma_{i}\right)\;=\;\frac{\Delta_{fus}H}{R}\left(\frac{1}{T_{m}}\;-\;\frac{1}{T}\right)\;$$

where *γ* is the activity coefficient of solute, Δ_fus_*H* is the molar melting enthalpy at melting temperature *T*_m_. The values of *T*_m_ and Δ_fus_*H* of metoprolol succinate have been determined, as shown in Section 2.1.

To calculate the solubility of a solute in a solvent, the activity coefficient γ can be expressed by the Wilson model [22], as shown in Equations (6) and (7):

(6) $$\ln\gamma_{2}\;=\;-\ln(x_{2}\;+\;\Lambda_{21}x_{1})\;+\;x_{1}\left[\frac{\Lambda_{21}}{x_{2}\;+\;\Lambda_{21}x_{1}}\;-\;\frac{\Lambda_{12}}{x_{1}\;+\;\Lambda_{12}x_{2}}\right]$$

(7) $$\ln\gamma_{1}\;=\;-\ln(x_{1}\;+\;\Lambda_{12}x_{2})\;+\;x_{2}\left[\frac{\Lambda_{12}}{x_{1}\;+\;\Lambda_{12}x_{2}}\;-\;\frac{\Lambda_{21}}{x_{2}\;+\;\Lambda_{21}x_{1}}\right]$$

The binary model parameters, Λ_ij_, in the Wilson model are expressed as [22]:

(8) $$\ln\Lambda_{\mathrm{ij}}\;=\;a_{\mathrm{ij}}\;+\;\frac{b_{\mathrm{ij}}}{T}$$

where Λ_ij_ is the Wilson parameter, and *a*_ij_ and *b*_ij_ are the temperature-independent parameters.

#### 2.4.3. NRTL Model

According to the NRTL model, the activity coefficient of the solvent can be calculated using Equation (9) as:

(9) $$\ln\gamma_{1}\;=\;x_{{}_{2}}^{2}\left[\frac{\tau_{21}G_{21}^{2}}{{\left(x_{1}\;+\;x_{2}G_{21}\right)}^{2}}\;+\;\frac{\tau_{12}G_{12}^{}}{{\left(x_{2}\;+\;x_{1}G_{12}\right)}^{2}}\right]$$

Here, the interaction parameter *G*_ij_ can be expressed as follows:

(10) $$G_{12}\;=\;\exp(-\sigma_{12}\tau_{12})$$

(11) $$G_{21}\;=\;\exp(-\sigma_{21}\tau_{12})$$

Here, *σ*_ij_ is a parameter relating to the non-randomness of the solution, which ranges from 0.2–0.47. In this study, the value *σ* was fixed at 0.3 in the regression analysis [24,25]. The temperature-dependent interaction parameter τij can be expressed as [26]:

(12) $$\tau_{\mathrm{ij}}\;=\;a_{\mathrm{ij}}\;+\;\frac{b_{\mathrm{ij}}}{T}$$

where *a*_ij_ and *b*_ij_ are two adjustable parameters relating to the interaction energy between the molecules i and j. These parameters are independent of temperature.

#### 2.4.4. Correlation Results

The measured solubilities of metoprolol succinate in the studied solvents were correlated using the above equations via non-linear regression analysis. The parameters of the different thermodynamic models were obtained and are listed in Table 3.

The obtained temperature-dependent binary interaction parameters were tested for their ability to predict the solubilities of metoprolol succinate in different solvents at all of the experimental temperatures. The average relative deviation (*ARD*%) values are shown in Table 4.

### 2.5. QSPR and DFT Studies

To understand the mechanism of dissolution, a simple quantitative structure–property relationship (QSPR) model between the physical properties of the alcohols and the solubilities of metoprolol succinate in these solvents was tested. The physical properties of the solvents and their linear regression coefficients with the solubility data are listed in the Supplementary Material (Tables S1 and S2).

**Figure 3:**
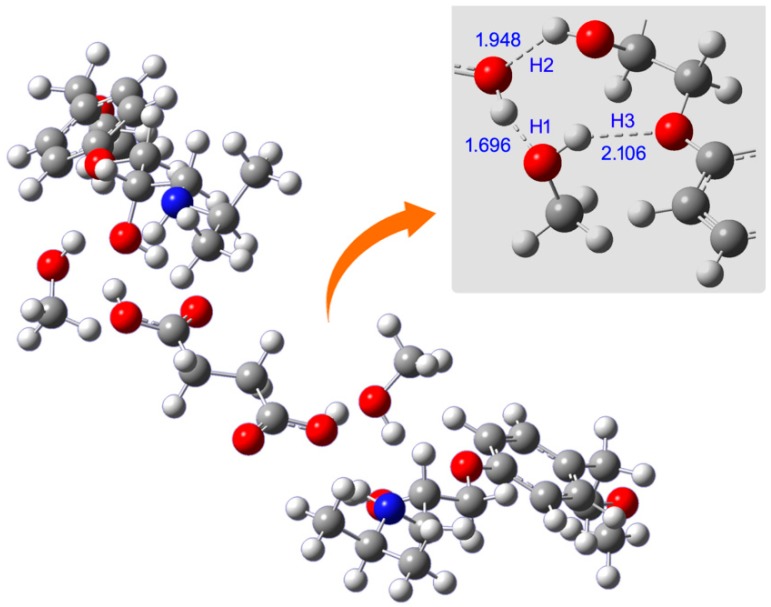
Interactions between metoprolol succinate and methanol; The dashed lines indicate hydrogen bonds of O–H with a distance (Unit: Å, 1 angstrom = 0.1 nm).

The structure and configuration of metoprolol succinate with alcohols are studied by using DFT calculations. The structure of metoprolol succinate was first optimized and this is shown in Figure S1 in the Supplementary Materials. The optimized structures of metoprolol succinate with ethanol, propanol, butanol, and isopropanol are also shown in Figures S2–S5, respectively. As an example, the optimized configuration of metoprolol succinate with methanol is shown in Figure 3. The hydrogen bonds (H1, H2, and H3) with the values of distance in the complex structure are shown in the figure. It was found that the hydrogen bonding played an important role in the solubility of metoprolol succinate in alcohol solvents. The hydrogen bond distances of metoprolol succinate with each alcohol are calculated and shown in Table 5.

## 3. Discussion

The solubilities of metoprolol succinate in alcohols, especially methanol, are much higher than those in acetone and ethyl acetate. The solubility of metoprolol succinate in methanol is about one order of magnitude higher than those in other alcohols at the same temperature. The solubility of metoprolol succinate in methanol was found to have the strongest positive dependency on the temperature, but the solubility of metoprolol succinate increased with increasing temperature for all solvents. Furthermore, the solubility of metoprolol succinate in the tested solvents decreased in the order methanol > ethanol > *n*-butanol > *n*-propanol > isopropanol > acetone > ethyl acetate. The solubility of metoprolol succinate in *n*-butanol was similar to that of *n*-propanol. On the other hand, for alcohols containing the same number of carbons, the solubilities in primary alcohols were higher than those in secondary alcohols (*n*-propanol > isopropanol). A similar trend was reported for the solubilities of sarpogrelate hydrochloride in alcohols [14].

For the thermodynamic functions of dissolution [27] of metoprolol succinate, as shown in Table 2, in all cases, Δ*G*_sol,apparent_ is positive, which is similar to the apparent enthalpy of dissolution of many drugs [28,29] reported in the literature. A lower value of Δ*G*_sol,apparent_ indicates a higher solubility of metoprolol succinate. The apparent enthalpies and entropies of dissolution of metoprolol succinate in all solvents are positive. This indicates that the dissolution of metoprolol succinate is endothermic.

In the thermodynamic modeling section, the modified Apelblat equation, Wilson model, and NRTL model were employed to correlate the solubilities of metoprolol succinate in different solvents. Results show that the calculated solubility values of metoprolol succinate in the seven solvents agreed well with the experimental values. The maximum value of ARD% was 5.29%, which was attained with the Apelblat equation for ethanol. The average ARD% values in the seven selected solvents for the Apelblat, Wilson, and NRTL models are 1.93%, 1.30%, and 2.44%, respectively. This means that the Wilson model showed the best performance for correlating the solubility data for metoprolol succinate in different solvents. Concerning the two-parameter activity coefficient models, the obtained ARD% values obtained from the NRTL model are a little larger than those of the Wilson model, especially for the systems of metoprolol succinate and ethanol or acetone. In their original paper, Renon and Prausnitz [23] related *σ* to the reciprocal of the coordinate number, which is the number of molecules connected to the reference molecule. For equilibrium calculations, the suggested range of the *σ* values is between 0.2 and 0.47, depending on the system and components involved. We found that no general value of *σ* could be found for all the systems studied. In this study, a global average *σ* value of 0.3 was used, which was typically chosen for the solubility modeling using the NRTL equation [24,25]. Notably, the *σ* value is treated as an additional adjustable parameter [24], and the NRTL equation gives the best correlation results. However, the results show that both the two-parameter activity coefficient equations can provide acceptable results. In general, the predicted solubility data from the Wilson equation showed excellent agreement with the experimental solubilities of metoprolol succinate in solvents at temperature ranging from (278.2 to 318.2) K at atmospheric pressure. The Wilson equation was adapted to correlate the solubility data with good results and could be easily extended to predict the solubility in the corresponding binary solvent mixtures [30,31].

The above experimental and thermodynamic results demonstrated that metoprolol succinate has a wide range of solubilities in alcohols, and its highest solubility was achieved in methanol. To understand the mechanism of dissolution, a simple QSPR model was tested. The results show that the coefficient between ln(*x*_exp_) and p*K*_a_ of the alcohols is about −0.96, indicating that the solubility of metoprolol succinate increases with increasing solvent acidity. For an organic salt of this drug, the acidic or basic properties of solvents play a dominant role in the dissolution process. In addition, the dielectric constant of the solvent is another important factor in the dissolving process, with a coefficient of 0.964. The dielectric constant of a solvent characterizes its chemical polarity [32]. The solubility of a polar drug increases as the solvent polarity increases. For an organic salt of a drug, as the polarity of the solvent increases, the intermolecular dipole or hydrogen bonging interactions become stronger, resulting in an increase in the solubility. The coefficients of the solubility data with Hansen solubility parameters (the parameters of basic, dipolar and hydrogen bonding with coefficients >0.90) also shows the same trend, as shown in Table S2 in the Supplementary Material. Thus, the primary QSPR results suggest that the polarity of alcohol solvents and their intermolecular hydrogen bonding interactions play an important role in the dissolution of metoprolol succinate.

However, the solubility values of metoprolol succinate in alcohol solvents were not decreased linearly with an increasing carbon number in alcohol molecules. For example, the solubility of metoprolol succinate was much higher in methanol, and it was higher in *n*-butanol than isopropanol. To understand the mechanism of dissolution on a molecular level, DFT calculations were performed. Figure 3 displays interactions between metoprolol succinate and methanol. The hydrogen bonds were built between methanol and metoprolol succinate according to the DFT calculations. It can be seen that the bond distances indicate that hydrogen bonds were the main intermolecular interactions in the case of metoprolol succinate with alcohol solvents [28]. As shown in the figure, the hydrogen bonds can occur between the H and O atoms in the molecular structures of metoprolol, succinic acid and methanol. The hydrogen bonds H1 and H3 are formed by methanol with succinic acid and metoprolol, respectively, while H2 represents the intramolecular hydrogen bond between succinic acid and metoprolol in the structure of metoprolol succinate. The results showed that the hydrogen bond H2 (with distance values between 1.95 and 2.00 Å) is quite stable in the complex structure. It was interesting to find that the hydrogen bond of H1 with distance of 1.696 Å only occurred between methanol and succinic acid, as shown in Table 3. This result is in accordance with the experimental data that the solubility of metoprolol succinate in methanol is much larger than in other alcohol solvents. The values of H1, H2, and H3 for *n*-butanol and *n*-propanol were very close, which correlates with their similar capabilities in the dissolution of metoprolol succinate. It can also been seen that the distance of the H3 bond of isopropanol (2.256 Å) is larger than that of *n*-butanol and *n*-propanol. Thus, isopropanol gave the lowest solubility for metoprolol succinate. It was reasonable that the sterically hindered isopropyl group in isopropanol would weaken its hydrogen bonding with metoprolol succinate, as shown in the optimized structures. Overall, the DFT results well illustrated that the solubility of metoprolol succinate in alcohols can be mainly attributed to hydrogen bonding interactions between metoprolol succinate and alcohol molecules.

## 4. Materials and Methods

### 4.1. Materials

Metoprolol succinate was obtained from Zhejiang Haixiang Co., Ltd. (Taizhou, Zhejiang, China) with a mass purity of 99.8%, which was checked by high-performance liquid chromatography (HPLC) (Agilent Technologies, Palo Alto, CA, USA). The compound was further recrystallized in methanol before use. The melting point of metoprolol succinate was determined using a differential scanning calorimeter (TA Q200) (TA Instruments, New Castle, DE, USA) [16]. The solvents isopropanol, *n*-butanol, methanol, ethanol, *n*-propanol, ethyl acetate, and acetone were of analytical grade and they were used without additional purification.

### 4.2. HPLC Analysis

The concentrations of metoprolol in solutions were determined using an Agilent 1260 HPLC system (Agilent Technologies, Palo Alto, CA, USA). An Agilent ZORBAX 80 A Extend-C18 column (5 µm, 4.60 × 150 mm) was used. The HPLC conditions were optimized, with detection being monitored at 280 nm. A mobile phase composed of pH 3.0 phosphate buffer and acetonitrile in a volume ratio of 2.5:7.5 was selected. The flow rate of the mobile phase was 1.0 mL min^−1^. A calibration curve with a regression coefficient of 0.9999 was obtained and used to determine the concentration of metoprolol succinate in solution.

### 4.3. Solubility Measurements

This process has been described in detail in our previous papers [33,34]. Briefly, an excess amount of metoprolol succinate was added to flasks with various pure solvents (isopropanol, *n*-butanol, methanol, ethanol, *n*-propanol, ethyl acetate, and acetone). The flasks were shaken in an SHKA4000-8CE incubator shaker (Thermo Scientific, Waltham, MA, USA) for more than 12 h to allow equilibrium. In this study, temperatures ranging from 278.2 to 318.2 K with a step of 5 K were tested. The temperature uncertainty of shaker was ±0.1 K. After equilibrium, the suspended solutions in flasks were allowed to settle for another 1 hr at selected temperatures. Then, the clear upper part of the solution was withdrawn carefully. The solution was filtered with a 0.45 µm membrane. The filtrate was diluted appropriately and determined by the HPLC condition as described above. All experiments were performed three times and the data are shown as the mean ± standard deviation (SD).

### 4.4. Thermal Analysis

A differential scanning calorimeter (TA Q200, New Castle, DE, USA) was used to determine the enthalpy of fusion and the melting temperature of metoprolol succinate. An accurately weighed solid sample (5.33 mg) was placed in a sealed aluminium pan with a vented lid to allow the dehydration of the sample. Then, it was heated under nitrogen flow (50 mL min^−1^) at a scanning rate of 10 °C min^−1^ from (10 to 300) °C. An empty aluminium pan was used as a reference. Three independent experiments were performed, and the data are shown as the mean ± standard deviation (SD).

### 4.5. Theoretical Calculations

For the theoretical calculations, all of the structures in the present calculations were optimized by hybrid B3LYP functional [35] combining with the 6-31G(d) basis set. Different geometries of pure species and metoprolol succinate–solvent complexes in the gas phase were optimized. All of the calculations were performed by using Gaussian09 software package [36].

## 5. Conclusions

The solubilities of metoprolol succinate in methanol, ethanol, *n*-propanol, isopropanol, *n*-butanol, ethyl acetate, and acetone were measured at temperatures ranging from 278.2 to 318.2 K. The solubility of metoprolol succinate decreased in the following order: methanol > ethanol > *n*-butanol > *n*-propanol > isopropanol > acetone > ethyl acetate. The solubility in all solvents increased with increasing temperature. The enthalpy of fusion and the melting point of metoprolol succinate were determined by DSC. The modified Apelblat equation, the Wilson model, and the NRTL model were compared for the correlation of solubility data. All three solubility models provide acceptable results with small ARD% values, but the Wilson equation gives the best results, having an average ARD% of 1.3%. Finally, DFT calculations based on the B3LYP level indicate that the solubility of metoprolol succinate in alcohol solvents can be mainly attributed to intra- and intermolecular hydrogen bonds in metoprolol succinate–solvent complexes.

## Figures and Tables

**Table 1:** Experimental mole fraction solubilities of metoprolol succinate, 10^3^∙*x*_exp_, in different solvents at temperatures *T* = (288.2 to 318.2) K under 0.1 MPa.^1^

*T*/K	Methanol	Ethanol	*n*-Butanol	*n*-Propanol	Isopropanol	Ethyl Acetate	Acetone
288.2	2.845 ± 0.068	0.435 ± 0.010	0.177 ± 0.006	0.165 ± 0.006	0.074±0.002	0.019 ± 0.002	0.061 ± 0.004
293.2	3.548 ± 0.096	0.559 ± 0.012	0.259 ± 0.001	0.258 ± 0.008	0.109 ± 0.002	0.028 ± 0.005	0.089 ± 0.002
298.2	4.741 ± 0.107	0.822 ± 0.015	0.377 ± 0.019	0.373 ± 0.006	0.160 ± 0.001	0.040 ± 0.003	0.130 ± 0.002
303.2	6.424 ± 0.228	1.047 ± 0.009	0.536 ± 0.024	0.548 ± 0.005	0.219 ± 0.003	0.058 ± 0.004	0.173 ± 0.004
308.2	8.745 ± 0.091	1.416 ± 0.050	0.788 ± 0.065	0.831 ± 0.019	0.316 ± 0.011	0.084 ± 0.006	0.234 ± 0.008
313.2	12.547 ± 0.012	2.175 ± 0.084	1.111 ± 0.085	1.240 ± 0.027	0.465 ± 0.027	0.118 ± 0.009	0.299 ± 0.003
318.2	16.631 ± 0.112	3.172 ± 0.098	1.567 ± 0.086	1.795 ± 0.012	0.659 ± 0.053	0.165 ± 0.008	0.425 ± 0.015

^1^ Standard uncertainty *u*(*T*) = 0.1 K; Relative Standard uncertainties *u*_r_(*p*) = 0.005, and *u*_r_(*x*) = 0.02.

**Table 2:** Thermodynamic parameters relative to the solution process of metoprolol succinate in different solvents at the mean harmonic temperature *T*_hm_ = 302.9 K under 0.1 MPa.^1^

Solvent	Δ*H*_sol,apparent_/(kJ·mol^−1^)	Δ*G*_sol,apparent_/(kJ·mol^−1^)	Δ*S*_sol,apparent_/(J·kJ^−1^·mol^−1^)
Methanol	45.87	12.63	109.74
Ethanol	50.11	17.14	108.87
*n*-Butanol	55.50	18.96	120.63
*n*-Propanol	60.48	18.88	137.34
Isopropanol	55.26	21.17	112.54
Ethyl acetate	54.94	24.59	100.20
Acetone	48.06	21.90	86.37

^1^ Standard uncertainties are *u*(*T*) = 0.1 K; Relative Standard uncertainties *u*_r_(*p*) = 0.005.

**Table 3:** The calculated parameters of the equations for solubilities in different solvent.

Solvent	Methanol	Ethanol	*n*-Butanol	*n*-Propanol	Isopropanol	Ethyl Acetate	Acetone
Modified Apelblat equation							
*A*	−632.7	−120.6	−133.5	−144.8	−238.9	−134.8	−117.7
*B*	23603.6	−1.76	−2.05	−2.31	4736.27	−2.08	−1.70
*C*	96.21	19.92	22.05	24.03	37.61	21.88	19.08
Wilson model							
*a*_12_	−17.95	−13.26	−15.27	−10.30	−23.11	−20.08	−24.62
*b*_12_	8001.6	2711.6	5797.5	2208.8	5882.2	6752.4	8799.2
*a*_21_	−35.33	0.41	−2.35	0.39	1.33	−2.00	−0.76
*b*_21_	9900.8	602.7	949.3	552.1	256.3	750.8	0.4
NRTL model							
*a*_12_	0.0687	0.7800	345.90	387.52	−0.0685	1.8082	0.1989
*b*_12_	−938.5	−1045.6	−1426.6	−1202.9	−736.6	−1213.6	−903.2
*a*_21_	5.41	4.16	21.02	19.00	13.10	6.59	10.73
*b*_21_	−110.5	−173.3	−7899.9	−7300.7	−2779.1	−972.6	−1293.4

**Table 4:** The average relative deviation (*ARD*%) of models for solubilities in different solvents.

Solvent	*ARD*%		
	Modified Apelblat	NRTL Model	Wilson Model
Methanol	1.50	1.59	0.85
ethanol	5.29	4.17	2.38
*n*-Butanol	0.47	0.92	0.44
*n*-Propanol	1.16	1.98	1.27
Isopropanol	1.28	1.29	1.17
Ethyl acetate	1.06	2.41	0.64
Acetone	2.73	4.71	2.34
Average	1.93	2.44	1.30

**Table 5:** The values of distance of hydrogen bonds (H1, H2, and H3) of metoprolol succinate in different solvents calculated by density functional theory (DFT).

Solvent	Bond Distance/Å		
	H1	H2	H3
Methanol	1.696	1.948	2.106
Ethanol	3.851	1.975	2.023
*n*-Butanol	3.870	1.998	2.051
*n*-Propanol	3.877	1.997	2.048
Isopropanol	3.865	1.960	2.256

## Supplementary Materials

The following are available online, Table S1: Experimental solubilities of metoprolol succinate, ln(*x*_exp,298K_), and the solvent property values used in the QSPR study; Table S2: The linear correlation coefficient values between solubility ln(*x*_exp,298K_) and each physical property. Figure S1: The optimized structure of metoprolol succinate as calculated by DFT; Figure S2: The optimized structure of metoprolol succinate with ethanol as calculated by DFT; Figure S3: The optimized structure of metoprolol succinate with *n*-propanol as calculated by DFT; Figure S4: The optimized structure of metoprolol succinate with *n*-butanol as calculated by DFT; Figure S5: The optimized structure of metoprolol succinate with isopropanol as calculated by DFT.
