# Supplementary material for: Measurements, Thermodynamic Modeling, and a Hydrogen Bonding Study on the Solubilities of Metoprolol Succinate in Organic Solvents
Source: Molecules. 2018 Sep 26;23(10):2469. doi: 10.3390/molecules23102469 (PMC6222524; doi:10.3390/molecules23102469)
Supplement: Supplementary file 1 [file molecules-23-02469-s001.pdf]

*Supplemental information*

# Experimental Measurements and Thermodynamic Modeling of the Solubilities of Metoprolol Succinate in Organic Solvents at Different Temperatures

**Jian Shen [1,*], Xianrui Liang [2] and Hao Lei [1]**

[1] School of Materials and Chemical Engineering, Ningbo University of Technology, Ningbo 315016, China
[2] College of Pharmaceutical Sciences, Zhejiang University of Technology, Hangzhou 310014, China
* Correspondence: shenjian0197@163.com; Tel.: +86-151-6854-9693

**Table S1.** Experimental solubilities of metoprolol succinate, ln($x_{\mathrm{exp,\ 298\ K}}$), and solvent property values used in QSPR study

| Property | Methanol | Ethanol | *n*-Butanol | *n*-Propanol | Isopropanol |
|---|---|---|---|---|---|
| ln($x_{\mathrm{exp,\ 298\ K}}$) | 1.5562 | −0.1960 | −0.9755 | −0.9862 | −1.8326 |
| *Physical properties* [a] | | | | | |
| Molecular weight (g/mol) | 32.04 | 46.07 | 74.12 | 60.1 | 60.1 |
| Density at 25 °C (g/mL) | 0.792 | 0.7893 | 0.81 | 0.803 | 0.786 |
| Viscosity at 25 °C (mPa·s) | 0.545 | 1.074 | 2.573 | 1.959 | 1.96 |
| Molar Volume ($cm^3$/mol) | 40.7 | 58.7 | 92 | 75.1 | 76.9 |
| Dipolar (D) | 1.69 | 1.69 | 1.66 | 1.68 | 1.66 |
| Acidity (p*K*a) | 15.5 | 15.9 | 16.1 | 16 | 16.5 |
| Refractive index | 1.3314 | 1.3611 | 1.3993 | 1.3870 | 1.3776 |
| Melting point (°C) | −98 | −114.14 | −89 | −127 | −89.5 |
| Boiling point (°C) | 64.7 | 78.29 | 117.7 | 97 | 82.6 |
| Dielectric Constant | 32.6 | 24.3 | 17.8 | 20.1 | 18.3 |
| *Hansen solubility parameters ($MPa^{1/2}$)* [b] | | | | | |
| Dispersion | 15.14 | 15.75 | 15.95 | 15.95 | 15.75 |
| Dipolar | 12.27 | 8.8 | 5.73 | 6.75 | 6.14 |
| Acidic | 17.18 | 16.98 | 13.09 | 15.34 | 14.52 |
| Basic | 14.52 | 11.25 | 9.41 | 9.82 | 9.2 |
| Hydrogen Bonding | 22.3 | 19.43 | 15.75 | 17.39 | 16.36 |

[a] [1] D.R. Lide, CRC Handbook of Chemistry and Physics, Internet Version 2005, CRC Press, Boca Raton, FL, (2005); [2] PubChem: https://pubchem.ncbi.nlm.nih.gov.

[b] A. Jouyban, Handbook of solubility data for pharmaceuticals, CRC Press.

**Table S2.** The linear correlation coefficient values between solubility ln($x_{exp,\ 298\ K}$) and each physical properties.

| | **Coefficients** |
|---|---|
| ln($x_{exp,\ 298\ K}$) | 1.000 |
| Molecular weight | −0.833 |
| Density at 25 °C (g/mL) | −0.136 |
| Viscosity at 25 °C (mPa·s) | −0.836 |
| Molar Volume | −0.854 |
| Dipolar | 0.773 |
| Acidity (p$K_a$) | −0.960 |
| Refractive index | −0.850 |
| Melting point (°C) | −0.071 |
| Boiling point (°C) | −0.601 |
| Dielectric Constant | 0.964 |
| Dispersion | −0.847 |
| Dipolar | 0.956 |
| Acidic | 0.733 |
| Basic | 0.978 |
| Hydrogen Bonding | 0.940 |

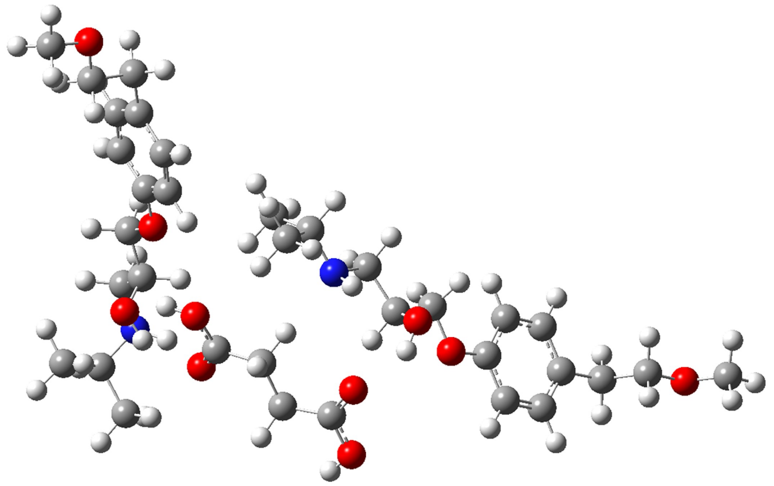

**Figure S1.** The optimized structure of metoprolol succinate calculated by DFT.

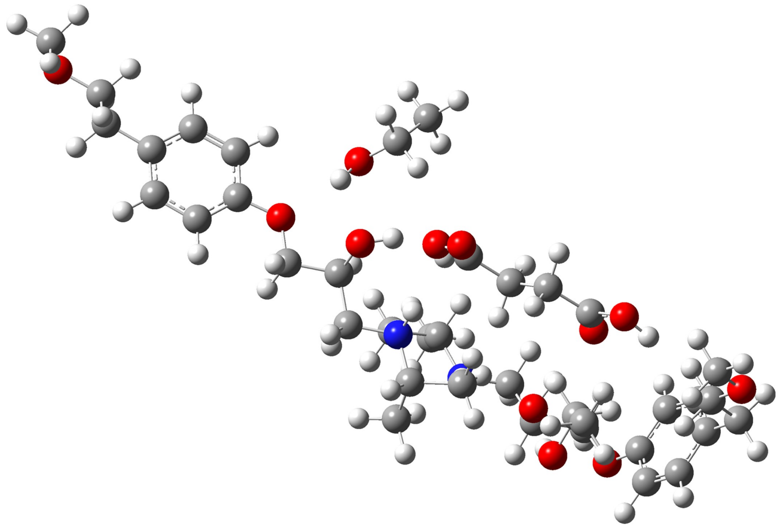

**Figure S2.** The optimized structure of metoprolol succinate with ethanol calculated by DFT.

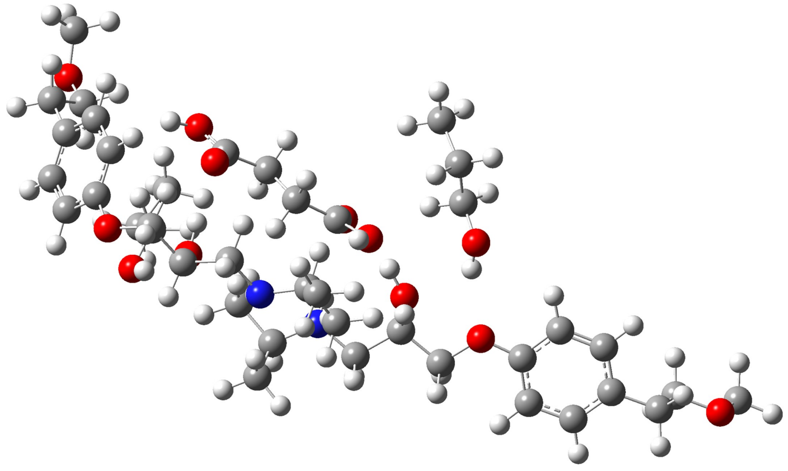

**Figure S3.** The optimized structure of metoprolol succinate with *n*-propanol calculated by DFT.

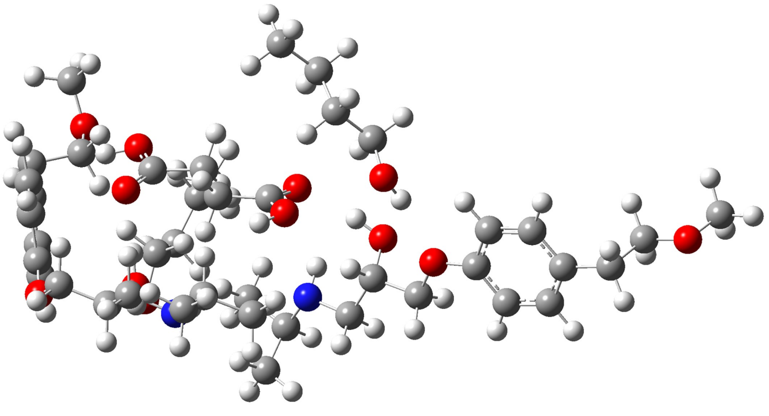

**Figure S4.** The optimized structure of metoprolol succinate with *n*-butanol calculated by DFT.

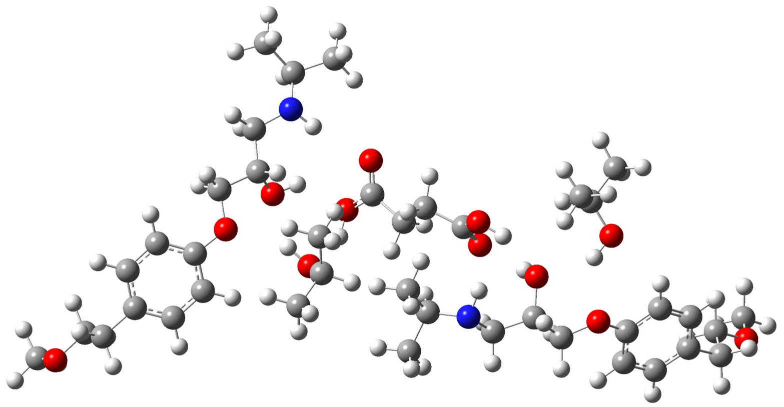

**Figure S5.** The optimized structure of metoprolol succinate with isopropanol calculated by DFT.
